# Neutrino communication, intergalactic transit, and Astrobiology

**DOI:** 10.6026/97320630018072

**Published:** 2022-02-28

**Authors:** Paul Shapshak

**Affiliations:** 1 Division of Infectious Diseases and International Health, Tampa General Hospital, Department of Internal Medicine, University of South Florida, Morsani College of Medicine, Tampa, FL 33606, USA

**Keywords:** Neutrino, communication, detection, transmission, electro-magnetism, signal, galaxy, paradox, astrobiology, astro virology, extra-terrestrial intelligence (ETI), Standard Model, boson, X-17, Glashow resonance, flavor, oscillations, weak force

## Abstract

This is a brief summary, snapshot, of a few issues that relate to possible communication with Extraterrestrial Intelligence (ETI) using neutrinos. Essentially, more research is required to better understand possible detection and communication with intelligent life (Astrobiology and ETI). Because of the possible scarcity of life in any single galaxy, to enhance the possibility of life detection, inter-galactic transit neutrinos necessitate consideration. Neutrino-based potential communications are inferred as the optimal mechanism or venue for detection of communications from ETI as well as sending communications to ETI. A paradox exists within this central theme. On the one hand, neutrino technology should be further developed and used to receive signals from or to send signals to ETI, because they transit inter-galactic distances. On the other hand, however, neutrinos have a very low cross-section interaction and are very difficult to detect. This concise Editorial incorporates several diverse research areas. Various issues are briefly and conjointly mentioned to inform the reader of multiple fields required towards a deeper understanding of astrobiology, astro virology, and ETI. This understanding is required for future advances, just as innovations in classical physics, quantum mechanics, particle physics, biophysics, chemistry, biochemistry, and molecular biology were required for the breakthroughs and advances in biology and virology, from the last century to the present - thus, the need for innovations and applications in neutrino particle physics research.

## Neutrinos and intergalactic communication:

Here some minimal history is provided, a few topics, and then a few examples of wide-ranging neutrino research specified which impact on Astrobiology, ETI, and communications. Neutrino physics was analyzed by Majorana in the late 1930's, which set the pace and cognizance of the importance of neutrino research in modern physics and cosmology. 1] It is noteworthy that Pasachoff and others, since 1979, proposed the use of neutrinos as an optimal means for interstellar communication. In addition, neutrinos were proposed as a means of telecommunications. 2,3,4 and 5] These were astute proposals since neutrinos are capable to traverse cosmic space-time distances without being blocked, where as electromagnetic radiation, though the primary means for communication are blocked to varying extents in the interstellar medium dust, clouds, nebulae, planets, and stars. 2,6] Since then, many articles reviewed and expanded on the use of neutrinos in communications, including their detection, production by natural processes, by artificial manufacture, and use for intergalactic interstellar communication. 7,8] Additional relevant unanticipated neutrino properties include the 2017 Biehl et al. description of conditions that would be indicative of tell-tale signs of neutrino production - the Glashow Resonance. The Glashow resonance is a process during which an energetic electron-flavor anti-neutrino interacts with an electron to produce a high energy weak force interaction W-negative boson, which then yields hadrons. 9] This process is summarized as follows.

Anti-ν_e_ + e → W-negative boson → hadrons at 6.3 PeV (peta-electron volts, the highest energy neutrino detected)

In addition, all three neutrino flavors were detected, and the neutrino source lies outside our galaxy. The specific extragalactic source was not yet identified. [10] Such processes are under study including types and modes of production. Additionally, it should be noted that muons may be damped in pion decay chains in the presence of magnetic fields, B, greater than 103 G (gauss). However, Glashow resonance is specific for the electron-anti-neutrino flavor and probably not suppressed under those conditions. Moreover, Glashow resonance does not occur for the other neutrino and anti-neutrino flavors (electron-, muon-, and tau-neutrino flavors as well as anti-muon- and anti-tau-neutrino flavors.) Thus, the significance of the Glashow resonance for the detection of a single specific neutrino flavor is indicated. [9] Additionally, Biehl et al. report that in their model, iron and silicon, which have neutron-rich nuclei, may further foster the anti-neutrino-electron Glashow resonance pathway [9]. Possibly, since some advanced Astro biological organisms and ETI may utilize iron and silicon in their repertoire of technologies, such considerations are not inconsequential. Processes such as these are sought after as means of widening the conditions for the tell-tale signs of neutrino production and possible ETI communication. In 2018, Hippke and colleagues produced benchmark comparisons of neutrinos with photons, electrons, protons, neutrons, Higgs, muon, and tau particles for their possible use in extraterrestrial communications. Various processes were discussed including comparing initial and operational costs. [11] Currently, we have insufficient terrestrial technological development to evaluate fully what advanced astro biological organisms and ETI civilizations may produce. An additional review published in 2020 supports the importance of neutrino research relevance for astrobiology and ETI. [8]. The challenge for intergalactic as well as interstellar communication and thus ETI detection, is to fully understand and extrapolate what artificial production and detection methods could be used for high energy particle physics and cosmology and whether they could be used feasibly and specifically for communications.

## Standard Model and beyond:

The Standard Model (SM) of particle physics is based on the work of many physicists, including Gel'man, Glashow, Weinberg, and Salam. The SM has been a steadfast bulwark for much of the 20th century and its validity secure. As we proceed, though, into the 21st century, various dissatisfactions and puzzles amidst the SM have been raised by Weinberg as well as others. [12,13] Theories beyond the SM are increasingly explored and developed. Insights from Albert Einstein and Richard Feynman illustrate considerations of physics beyond the SM. Albert Einstein asked the question, whether there is a deeper underlying reality for quantum mechanics than was understood in his day. Later, Richard Feynman raised that question again and added that no-one understood quantum mechanics. [14,15] This paper proposes that the concerns and research beyond the SM, involving neutrinos, bosons (including Higgs bosons), Dark Matter, Dark energy, etc., essentially, are addressing these questions. A brief example of this over-arching enigma in quantum mechanics is that anew boson, a possible fifth force, has been proposed. This surprising preliminary finding was made during the last few years from Debrecen, Hungary, and purports discovery of a light boson, X-17.So far, the same laboratory confirmed its own results. [16] Additionally, independent confirmation is required, and Conseil European pour la Recherche Nucleaire (CERN) has proposed to do this. [17]

## Neutrino:

Neutrinos commenced production and pervaded the expanding universe, one second after the Universe's Big Bang. In contrast, the cosmic microwave background of electromagnetic radiation was produced 380,000 years after the Big Bang. Neutrinos account for 0.5% of the Universe's density and have been produced unceasingly since the Big Bang. [18,19] In the last several decades, many large-scale scientific projects address these vital issues, and the importance of neutrinos has come to the fore. In this regard, several neutrino properties were reviewed by Rajasekaran and colleagues. The value of neutrinos was also discussed including their impact on communication - intergalactic and interstellar - and how such efforts comprise methods for neutrino production and detection. [7,8,20 & 21].

## Astrobiology:

Hypothetical astrobiology and exobiology are reviewed previously. [7,22]. One fundamental question is what caused terrestrial divergent chiralities among racemic mixtures of amino acids and carbohydrates? Are there differing Astrobiological models whereby chirality could be explained using endogenous generators vs. exogenous sources of chiralities and enantiomeric selection? One model for exogenous sources of biologically relevant compounds is whether amino acids could survive their in-fall to Earth inside meteorites and thus possibly impact terrestrial incipient biochemistry. Conversely, are there fundamental mechanisms for chirality selection in nature - i.e., in terrestrial nature? Nevertheless, the problem is impacted fundamentally by neutrinos. Da Motta analyzed the ideas and ramifications of chirality in nature and its impact on neutrinos. They discuss the central issues of sub-atomical particle spin and chirality, illustrating variances among quarks, bosons, and fermions, including neutrinos. [23] At the next level of analysis, Bargueno and de Tudela address the possibility that supernova neutrinos may influence molecular chirality in supernova remnants and their nearby clouds. Chiral discriminatory mechanisms due to neutrinos are described that could result in molecular chiral selection. [24] Inter related, in support of externally influenced biochemistry, Boyd and colleagues produced scenarios by which the L-enantiomer amino acids are produced in meteorites at some unspecified distances from Wolf-Rayet stars, cooling neutron stars, supernovae, magnetars, etc. [15] Consequently, it should be noted that the Sun, like any star, produces neutrinos that bathe the terrestrial environment, and neutrinos possibly could also be associated with dissymmetric chiral selection prior to and during the origin and evolution of life. [26] Terrestrial natural selective processes may have been associated with the selection of enantiomeric amino acids, sugars, and nucleotides. For example, selective adsorption experiments produced chiral amplification of amino acids in vermiculite clay. [27] In addition, where diastereomeric amino acids occur, enzymatic means can be utilized by organisms to produce L-amino acids. Accordingly, D, L-diamino-pimelic acid is converted to L-lysine in bacteria [28]. Nonetheless, terrestrially, although L-amino acids predominate among amino acids, conversely, D-sugars predominate among sugars.

However, D-amino acids are crucial for terrestrial life. Bacteria, closer to the origin of life than eukaryotes, contain D-amino acids (serine aspartic acid, and glutamic acid) in their cell walls for protection against exo-proteases produced by competitive bacteria. Vibrio cholerae releases D-amino acids into its environment as well as incorporating D-amino acids into its cell wall for self-protection from conditions of low osmolarity and from other bacteria. D-arginine promotes the growth of Vibrio cholerae. Additionally, and surprisingly, D-amino acids are found in soil, lakes, rivers, and oceans, as well as in some cells of eukaryotes; prokaryotes can survive under such conditions when otherwise starved. D-amino acids are utilized in the nervous systems and endocrine systems of eukaryotes, including D-serine, D-cysteine, D-alanine, D-aspartic acid, and N-methyl-D-aspartic acid. Finally, D-amino acids do slow protein production by ribosomes, and L-amino acids are, naturally, optimal; however, one should not accede to the simplistic notion that the absolute absence of D-amino acids was required for the origin and continued evolution of prokaryote and eukaryote terrestrial life. [22,27-31-check with author] The impact of neutrinos on L-amino acid survival in asteroids may be of relevance to some extraterrestrial origins of life, as well as for the terrestrial origin of life. [26]

## Conclusions:

Finding extra-terrestrial signals from hypothesized extraterrestrial galactic and extra-galactic ETI civilizations has been considered since the early 20th century. Multiple fields influence possible detection and communication using neutrinos, in addition to electromagnetic signaling. Neutrino artificial production and detection methods are under development, aimed at high energy particle physics and cosmology, which could be used for neutrino communications. [2,6] However, confronting astro biological ETI is many-sided, because of the assorted methods of manufacture, transmission, and range of signals, and the interstellar and intergalactic space-time magnitudes involved. [19] It is critical that we strengthen our understanding of whatever types of ETI, astrobiology, and astro virology may exist, preceding any possible encounter, no matter how unlikely. Foundations of global safety, health, and survival depend on it. The ancient Greek historian, Thucydides, pointed out the safety risks of foreign power contacts some 2,433 years ago, let alone problems to encounter ETI. [32-check with author] Technological terrestrial civilization is at a very early stage and much work and progress are required.

## References

[1] https://link.springer.com/article/10.1007/BF02961314.

[2] https://ui.adsabs.harvard.edu/.

[3] https://www.sciencedirect.com/science/article/abs/pii/0094576579901577?via%3Dihub.

[4] https://www.jstor.org/stable/24537195.

[5] https://pubmed.ncbi.nlm.nih.gov/17770504/.

[6] https://www.annualreviews.org/doi/10.1146/annurev.astro.39.1.511.

[7] https://baas.aas.org/pub/2020n7i009/release/1.

[8] https://arxiv.org/pdf/2007.05736.pd.

[9] https://arxiv.org/pdf/1611.07983.pdf.

[10] https://arxiv.org/abs/1405.5303.

[11] https://arxiv.org/pdf/1711.07962.pdf.

[12] https://cds.cern.ch/record/799984/files/0401010.pdf.

[13] https://www.tau.ac.il/.

[14] https://journals.aps.org/pr/abstract/10.1103/PhysRev.47.777.

[15] https://arxiv.org/abs/0804.3348.

[16] https://journals.aps.org/prc/abstract/10.1103/PhysRevC.104.044003.

[17] https://link.springer.com/article/10.1140/epjc/s10052-020-08725-x.

[18] https://journals.aps.org/prd/abstract/10.1103/PhysRevD.98.081301.

[19] https://academic.oup.com/ptep/article/2020/8/083C01/5891211.

[20] https://arxiv.org/pdf/1606.08715.pdf.

[21] https://link.springer.com/article/10.1007/s12043-000-0080-7.

[22] https://link.springer.com/book/10.1007/978-3-030-29022-1.

[23] https://iopscience.iop.org/article/10.1088/1742-6596/1558/1/012014.

[24] https://link.springer.com/article/10.1007/s11084-006-9060-3.

[25] https://iopscience.iop.org/article/10.3847/1538-4357/aaad5f.

[26] https://www.annualreviews.org/doi/10.1146/annurev-nucl-011921-061243.

[27] https://pubs.rsc.org/en/content/articlelanding/2011/CP/C0CP01388A.

[28] https://library.princeton.edu/databases/subject/dissertationstheses.

[30] https://academic.oup.com/nar/article/47/4/2089/5231803.

